# Unveiling the Biodiversity of Deep-Sea Nematodes through Metabarcoding: Are We Ready to Bypass the Classical Taxonomy?

**DOI:** 10.1371/journal.pone.0144928

**Published:** 2015-12-23

**Authors:** Antonio Dell’Anno, Laura Carugati, Cinzia Corinaldesi, Giulia Riccioni, Roberto Danovaro

**Affiliations:** 1 Department of Life and Environmental Sciences, Polytechnic University of Marche, Via Brecce Bianche, 60131 Ancona, Italy; 2 Stazione Zoologica Anton Dohrn, Villa Comunale, Naples, Italy; Università di Genova, ITALY

## Abstract

Nematodes inhabiting benthic deep-sea ecosystems account for >90% of the total metazoan abundances and they have been hypothesised to be hyper-diverse, but their biodiversity is still largely unknown. Metabarcoding could facilitate the census of biodiversity, especially for those tiny metazoans for which morphological identification is difficult. We compared, for the first time, different DNA extraction procedures based on the use of two commercial kits and a previously published laboratory protocol and tested their suitability for sequencing analyses of 18S rDNA of marine nematodes. We also investigated the reliability of Roche 454 sequencing analyses for assessing the biodiversity of deep-sea nematode assemblages previously morphologically identified. Finally, intra-genomic variation in 18S rRNA gene repeats was investigated by Illumina MiSeq in different deep-sea nematode morphospecies to assess the influence of polymorphisms on nematode biodiversity estimates. Our results indicate that the two commercial kits should be preferred for the molecular analysis of biodiversity of deep-sea nematodes since they consistently provide amplifiable DNA suitable for sequencing. We report that the morphological identification of deep-sea nematodes matches the results obtained by metabarcoding analysis only at the order-family level and that a large portion of Operational Clustered Taxonomic Units (OCTUs) was not assigned. We also show that independently from the cut-off criteria and bioinformatic pipelines used, the number of OCTUs largely exceeds the number of individuals and that 18S rRNA gene of different morpho-species of nematodes displayed intra-genomic polymorphisms. Our results indicate that metabarcoding is an important tool to explore the diversity of deep-sea nematodes, but still fails in identifying most of the species due to limited number of sequences deposited in the public databases, and in providing quantitative data on the species encountered. These aspects should be carefully taken into account before using metabarcoding in quantitative ecological research and monitoring programmes of marine biodiversity.

## Introduction

Field and theoretical studies increasingly argue that biodiversity influences ecosystem functions that are responsible for the production of natural goods and services for the entire biosphere (e.g., biomass production and nutrient regeneration) [1]. This appears to be particularly evident in the deep sea, where positive interactions (e.g., facilitation) apparently dominate among interspecific ecological interactions [2]. Thus, the ability to assess biodiversity over large spatial and temporal scales is crucial for a better understanding of the potential consequences of species loss on ecosystem functioning.

Deep-sea ecosystems cover ca. 65% of the surface of the globe and host a large fraction of the global biodiversity [3,4]. In benthic deep-sea ecosystems, nematodes, which account for >90% of the total metazoan abundances [4,5] are probably the best model organisms for exploring the relationship between biodiversity and ecosystem functioning [2,6]. Nematodes have been hypothesised to be hyper-diverse, and potentially representing one of the most diverse animal phyla [7], but at present, their biodiversity is still largely unknown. Despite many nematode taxa are morphologically highly diverse [8], the diversity at genus and species level often remains under-appreciated being time consuming and requiring highly specialised taxonomic expertise [9,10]. In addition, the use of morphological criteria can prevent the correct identification of nematode species since some of them show phenotypic plasticity and single morpho-species can hide cryptic diversity [11,12–14]. Molecular methods can solve these uncertainties and accelerate the difficult morphological identification of marine nematode species, thus allowing us to address key questions on cosmopolitanism, cryptic biodiversity and phylogeny [15,16]. DNA barcoding, based on the analysis of nuclear and mitochondrial genes, has been proposed since a decade for the assessment of the diversity of marine nematodes [17,18]. Molecular markers such as the nuclear small subunit and large subunit ribosomal RNA genes (i.e. 18S rRNA, 28S rRNA) and the mitochondrial cytochrome oxidase subunit 1 gene (COI) have been selected for nematode barcoding studies [10,18,19]. Linking molecular and morphological identification of nematode species can overcome taxonomic uncertainties and create a link between ecological/morphological investigations and community-based DNA analyses [17–22]. However, standard barcoding approaches based on Sanger sequencing are not appropriate for large-scale ecological investigations. In the last years, we are witnessing the rise in the use of High-Throughput Sequencing (HTS) platforms for the study of biodiversity of tiny metazoans, including nematodes, using homologous gene regions [23–27]. Nevertheless, the application of molecular tools to study benthic deep-sea nematodes is still in its infancy [20,28], partly due to the difficulty in collecting deep-sea nematodes and amplifying their DNA [29]. Thus there is an urgent need to identify reliable procedures for the recovery of DNA from marine nematodes suitable for sequencing analyses. Another critical issue is to estimate the number of nematode species and their relative proportion in a sample from the number of Operational Clustered Taxonomic Units (OCTUs) obtained from HTS [26,30,31]. Terrestrial nematodes have a number of rRNA gene copies which can vary dramatically between species [32], hampering the possibility to correlate the number of OCTUs with the number of individuals. This issue has never been investigated in marine nematode assemblages.

In this study we compared, for the first time, different procedures for the recovery of DNA from marine nematode cultures and free-living nematodes collected from both coastal and deep-sea sediments suitable for Sanger and HTS analyses. Once extraction and amplification conditions of DNA were optimised, we investigated the reliability of Roche 454 sequencing analyses for assessing the biodiversity of deep-sea nematode assemblages (collected at two different benthic deep-sea sites) previously morphologically identified. Finally, intra-genomic variation in 18S rRNA gene repeats was investigated for different deep-sea nematode morphospecies in order to assess the influence of polymorphisms on nematode biodiversity estimates.

## Materials and Methods

### Ethics statement

All of the field activities were approved by the local national authorities (Spanish and Italian Ministry). The sampling areas were not privately owned or protected in any way, and no endangered or protected species were involved in this study.

### Sample collection and processing

Three cultured species of marine nematodes and free-living nematodes collected from coastal and deep-sea sediments were used to identify the most efficient procedure for the recovery of DNA suitable for Sanger and HTS analyses. Nematodes belonging to the species *Diplolaimelloides oschei*, *Pellioditis marina* and *Plectus sp*. were grown in Petri dishes containing 0.75% bacto-agar medium and maintained under controlled conditions at 20°C in the dark [33]. Benthic shallow-water nematodes were extracted from sediment samples collected using manual corers at ca. 2 m depth in the central Adriatic Sea (Mediterranean Sea). Deep-sea nematodes were recovered from sediment samples collected using a NIOZ-type box-corer in the NW Mediterranean Sea (Gulf of Lions) and in the Central Mediterranean Sea (Sicily Channel) both at ca. 500 m depth. After collection, sediment samples were stored without the use of any preservative at −20°C until further processing. Nematodes were recovered from the sediments using a 20 μm mesh net and then the fraction retained on the sieve was resuspended and centrifuged 3 times using Ludox HS40 (density 1.31 g cm^−3^) [34]. After centrifugation, all meiobenthic animals were sorted under a stereomicroscope. Then, each nematode was picked by using a sterile needle, and temporarily mounted on a slide using a drop of autoclaved MilliQ water and covered with a cover slip. Each nematode has been identified under light microscope to the genus or species-level when possible (indicated as sp1, sp2, sp3, etc., due to the presence of several unknown deep-sea species) according to previous studies [9,35] and the recent literature dealing with new nematode genera and species (i.e. NeMys database) [10]. After identification, each specimen was carefully picked from the slide and placed into a 2 ml sterile tube until further molecular analysis. Microscopic observations revealed that the storage at -20°C does not alter the morphological features used for taxonomic classification.

### Morphological and molecular analyses on single nematodes

To test for DNA extraction efficiency, different protocols were applied on single nematodes belonging to the same species and with a similar size extracted from the deep-sea and coastal sediments, and from cultures. Among free-living nematodes, individuals of *Sphaerolaimus uncinatus* were selected because this is one of the few formally described deep-sea nematode species [5] and it is distributed across different benthic habitats and ecosystems [10]. A total of 60 individuals, including: i) 10 organisms belonging to each of the three different species of cultured nematodes, ii) 15 individuals from benthic shallow systems and iii) 15 from deep-sea sediments, were processed and used for assessing DNA extraction efficiency. DNA from a single nematode was extracted using three different methods: i) a physical-chemical procedure (hereafter defined as “NaOH procedure”), ii) a commercial kit for tissue DNA extraction (QIAGEN DNeasy Blood & Tissue Kit), and iii) a commercial kit for DNA extraction from soils (PowerSoil DNA Isolation Kit, MoBio). The physical-chemical procedure was based on a method previously utilised for DNA extraction from nematodes [21] with slight modifications. Briefly, nematodes were added to 20 μL of 0.25 M NaOH and incubated overnight at -20°C. After incubation samples were heated at 60°C for 3 h followed by an incubation at 99°C for 3 min and then 4 μL HCl (1M), 10 μL Tris-HCl (0.5M, pH 8.0) and 5 μL Triton X-100 (2%) were added. Samples were again incubated at 99°C for 3 min and then stored at −20°C until further analysis (i.e. PCR amplification). DNA extraction from individual nematodes using the commercial kits (hereafter defined as QIAGEN kit and MoBio kit) was carried out following the instructions provided by the manufactures. After extraction, DNA was analysed fluorometrically using a NanoDrop ND-3300 Fluorospectrometer and SYBR Gold (Invitrogen) as fluorochrome. The fluorescence of DNA in the sample was converted into concentrations using calibration curves obtained from standard solutions of calf thymus DNA (from 2 to 50 pg μL^-1^). Differences in DNA concentrations obtained by using the different extraction procedures were checked by using the Mann—Whitney U test. To assess whether the extracted DNA was amplifiable, PCR analyses of DNA from single nematodes were carried out using the primer pairs Nem_18S_F (5’-CGCGAATRGCTCATTACAACAGC-3’) and Nem_18S_R (5’-GGGCGGTATCTGATCGCC-3’) [22] suitable for Sanger sequencing (amplicon length 900 bp) which is commonly used for phylogeographic and cryptic diversity analyses of nematodes. Details on PCR analyses are reported in the Appendix contained in S1 File. Sanger sequencing analyses were carried out by MACROGEN sequencing service (Macrogen Inc., Europe). Since the procedure based on the QIAGEN kit applied on deep-sea nematodes investigated in the present study provided DNA which was always successfully amplified when compared to the NaOH procedure (see the result section), all subsequent molecular analyses carried out on deep-sea nematode assemblages were based on the use of the QIAGEN kit.

### Morphological and molecular analyses on deep-sea nematode assemblages

To test the consistency and reliability of metabarcoding, nematodes extracted from deep-sea sediments were randomly picked, identified to species level (Tables A and B in S1 File) and then pooled before DNA extraction. DNA extraction was carried out on two assemblages of 10 and 100 individuals recovered from sediment samples collected in the NW Mediterranean and Central Mediterranean, respectively. Once extracted and purified, DNA was amplified using the primer pairs SSUF04 (5’-GCTTGTCTCAAAGATTAAGCC-3’) and SSUR22 (5’–CCTGCTGCCTTCCTTGGA-3’) [36] targeting the 18S rRNA gene and suitable, in term of amplicon length (450 bp), for metabarcoding analyses, according to the needs of the sequencing platforms utilised in the present study (see below). PCR reactions were carried out using the conditions and thermal protocol described in the Appendix contained in S1 File, but using ca. 1 ng DNA as template. Sequencing analysis was performed on a Roche 454 GS FLX Titanium platform by using Lib-L kit, by MACROGEN sequencing service (Macrogen Inc., Korea).

### Intra-genomic variability of 18S rRNA gene repeats within deep-sea nematodes

To investigate the intra-genomic variability of 18S rRNA gene repeats in marine nematodes, 5 different morphospecies have been collected from deep-sea sediments of the Central Mediterranean Sea, and DNA was extracted from each specimen using the QIAGEN kit. The 18S rRNA gene was amplified using the primer set SSUF04 (5’-GCTTGTCTCAAAGATTAAGCC-3’) and SSUR22 (5’–CCTGCTGCCTTCCTTGGA-3’) [36] and sequenced on the Illumina MiSeq platform (2 × 300 bp reads). The Illumina MiSeq platform has been selected because it has a sequencing error rate lower than Roche 454 [37], thus providing a more robust view of the actual variability of 18S rRNA gene repeats. Illumina MiSeq sequencing analysis was carried out by LGC Genomics (Germany).

### Bioinformatic analyses

#### Sanger sequences

Sequences obtained (both forward and reverse) by the Sanger procedure were manually checked using BioEdit v.7.2.0. Similarity search analysis of these sequences were performed against the GenBank database using MEGABLAST with default parameters.

#### Roche 454 data

Reads obtained by Roche 454 sequencing were processed using three different bioinformatic pipelines: i) OCTUPUS [24], ii) AmpliconNoise [38], as implemented in Mothur [39] and iii) QIIME [40]. Using OCTUPUS, after a quality check of sequences using Lucy [41] with default parameters and screening for a minimum length of 200 bp, sequences were clustered in OCTUs (Operational Clustered Taxonomic Units) both at 97% and 99% similarity to generate a list of consensus OCTUs. The obtained OCTUs were then BLAST-matched against the nucleotide GenBank and SILVA NR 119 databases. Taxonomic assignments for each OCTU were derived from the top-scoring BLAST match (exhibiting >90% pairwise identity). All OCTUs were analysed for the presence of putative chimeras using a frequency and length dependent algorithm incorporated into the OCTUPUS pipeline. All OCTUs identified as chimeras were removed from the analysis.

The same reads obtained from Roche 454 sequencing were also analysed using the AmpliconNoise [38], as implemented in Mothur [39], to remove 454 sequencing errors and PCR single base errors using default settings. Cleaned reads were analysed with Mothur using Perseus [38] to detect chimeras and the furthest neighbour method for clustering. OCTUs obtained at 97% and 99% clustering thresholds were BLAST-matched against the nucleotide GenBank and SILVA NR 119 databases and the top-scoring BLAST hit (exhibiting >90% pairwise identity) was used for taxonomic assignment.

Finally, the raw reads obtained from Roche 454 sequencing were analysed using QIIME pipeline [40]. Screening for a minimum length of 200 bp was performed by using split.libraries. Sorting, chimera detection and OCTUs picking were performed by using the USEARCH tool [42] implemented within the QIIME pipeline [40]. OCTUs obtained at 97% and 99% clustering thresholds were BLAST-matched against the Genbank and SILVA NR 119 databases and the top-scoring BLAST hit (exhibiting >90% pair-wise identity) was used for the taxonomic assignment.

A χ^2^-test with Yates continuity correction was used to check for the presence of statistical differences between the relative contributions of OCTUs assigned to nematode genera by metabarcoding and the relative contribution of individuals of the same genera identified by morphological analysis.

#### Illumina MiSeq data

QIIME pipeline was used to analyse the Illumina MiSeq sequences obtained from the five nematode morpho-species. Screening for a minimum length of 200 bp was performed by using the PRINSEQ tool [43]. Sorting, chimera detection and OCTUs picking were performed by using the USEARCH tool [42] implemented within the QIIME pipeline [40]. OCTUs obtained at 97% and 99% clustering thresholds were BLAST-matched against the GenBank database and the top-scoring BLAST hit (exhibiting >90% pairwise identity) was used for the taxonomic assignment.

OCTUs distribution among different taxonomic groups identified by using both Roche 454 and Illumina MiSeq sequencing were analysed by using MEGAN software [44], with default parameters.

## Results

### Recovery of DNA from single nematodes

**Fig 1:**
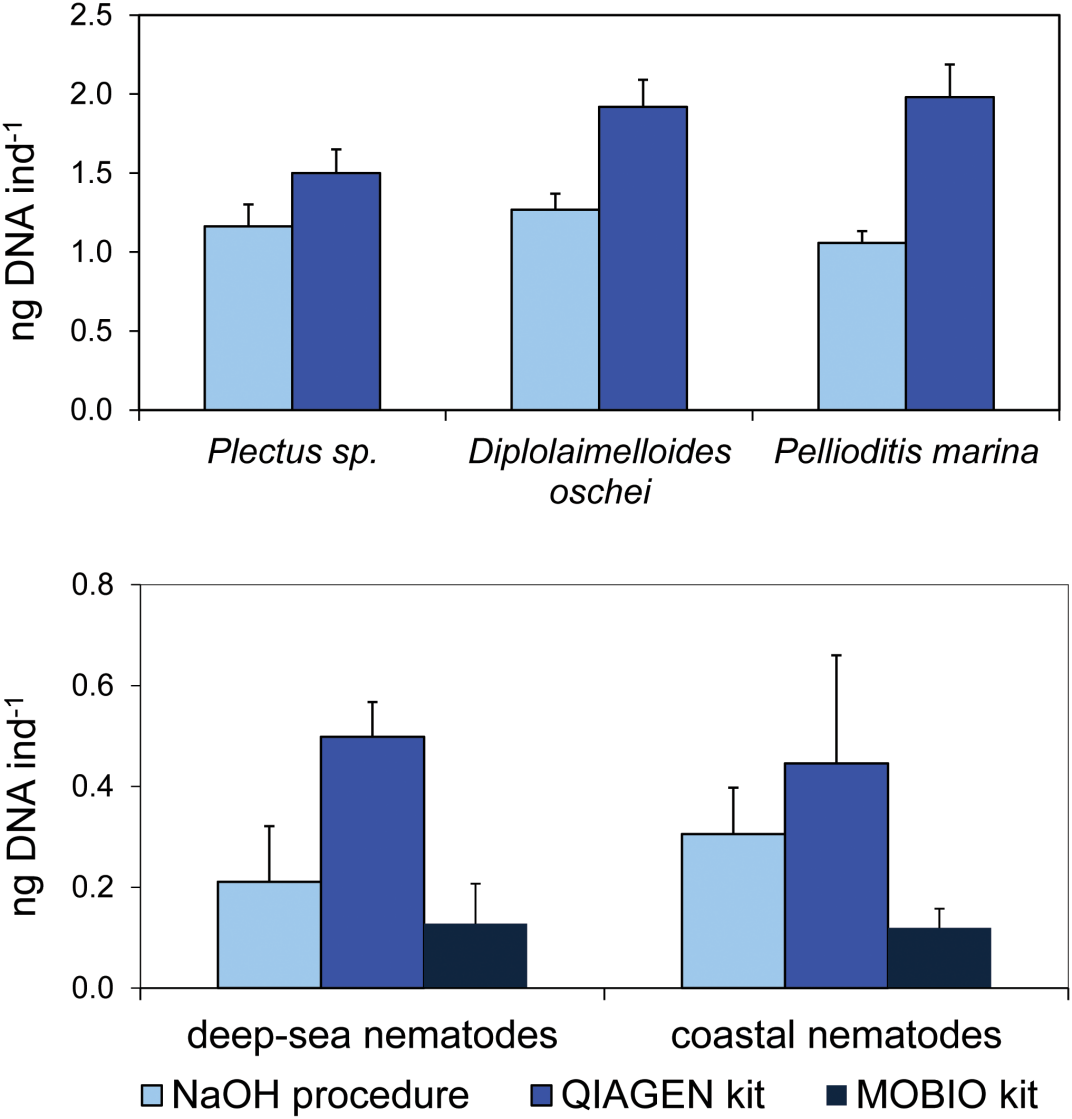
Comparison between DNA concentrations obtained by different DNA extraction procedures from free-living and cultured nematodes. Upper panel: comparison of DNA concentrations obtained using NaOH and QIAGEN kit extraction procedures from cultured nematodes (i.e. *Plectus* sp., *Diplolaimelloides oschei* and *Pellioditis marina*). Lower panel: comparison of DNA concentrations obtained using NaOH procedure, QIAGEN and MoBio kits from coastal and deep-sea nematodes. Mean (n = 5) and standard deviations are shown. The MoBio kit has not been used on cultured nematodes since they were not extracted from the sediment.

DNA concentrations of cultured nematodes extracted using the QIAGEN kit were significantly higher than those obtained using the NaOH procedure (p<0.01; Fig 1). The QIAGEN kit and the NaOH procedure provided DNA concentrations from coastal nematodes 3–4 fold higher than using the MoBio kit (Fig 1). DNA concentrations of nematodes collected from deep-sea sediments were significantly higher using the QIAGEN kit than using the two other DNA extraction procedures. Although DNA extracted from coastal nematodes with both the commercial kits and the NaOH procedure resulted in a successful amplification of 18S rRNA gene (Figure A in S1 File), the success of PCR amplification of DNA obtained from deep-sea nematodes by using the NaOH procedure was highly stochastic (even after several modifications including the addition of bovin serum albumin (BSA) to the reaction mix and tuning the PCR thermal protocol; Figure B in S1 File). This was not the case for the QIAGEN and MoBio kits, which always provided consistent and highly reproducible results in terms of 18S rRNA amplicons suitable for Sanger and HTS analyses (Figures B and C in S1 File).

### Biodiversity assessment: comparison between morphological vs. molecular approaches

The list of samples analysed by using Sanger, Roche 454 and Illumina MiSeq sequencing is reported in Table 1.

**Table 1:** List of samples analysed by using Sanger, Roche 454 and Illumina MiSeq sequencing.

	Samples	Number of reads	Number of reads after trimming	Pipeline	Database
**Sanger**	15 deep-sea nematodes	na	na	na	GenBank
	15 shallow-water nematodes	na	na	na	GenBank
	30 cultured nematodes	na	na	na	GenBank
**Roche 454**	10 deep-sea nematodes	56851	54949	OCTUPUS	GenBank and Silva
			1484	Mothur	GenBank and Silva
			23390	QIIME	GenBank and Silva
**Roche 454**	100 deep-sea nematodes	61647	58186	OCTUPUS	GenBank and Silva
			3518	Mothur	GenBank and Silva
			24747	QIIME	GenBank and Silva
**Illumina MiSeq**	*Halichoanolaimus sp1*	237034	2526	QIIME	GenBank
	*Sabatieria sp1*	157511	2282	QIIME	GenBank
	*Metachromadora sp1*	232452	3900	QIIME	GenBank
	*Viscosia sp1*	67677	2200	QIIME	GenBank
	*Thoracostoma sp*.	195381	2190	QIIME	GenBank

The database against which the BLAST search has been performed, the number of raw reads obtained, the number of reads obtained after trimming (sorting and chimera removal) and the bioinformatic pipelines utilised for data analysis are also reported. na = not applicable.

#### Analyses on single nematodes

The analysis of Sanger sequences obtained from each individual of cultured nematodes, based on a similarity search on GenBank, confirmed the morphological identification. Similarity search of Sanger sequences obtained from each individual of deep-sea nematodes morphologically classified as *Sphaerolaimus uncinatus* indicated 93% similarity with both *Sphaerolaimus hirsutus* and *Terschellingia longicaudata*. The similarity found with *T*. *longicaudata* could be explained considering that this species has a sister relationship with *S*. *hirsutus* (e.g., 18S rDNA sequences of *T*. *longicaudata* from Ras al Barr- Bahrain- are 99% identical with 18S rDNA sequences of *S*. *hirsutus*) [16].

#### Analyses on deep-sea nematode assemblages from the NW Mediterranean Sea

**Fig 2:**
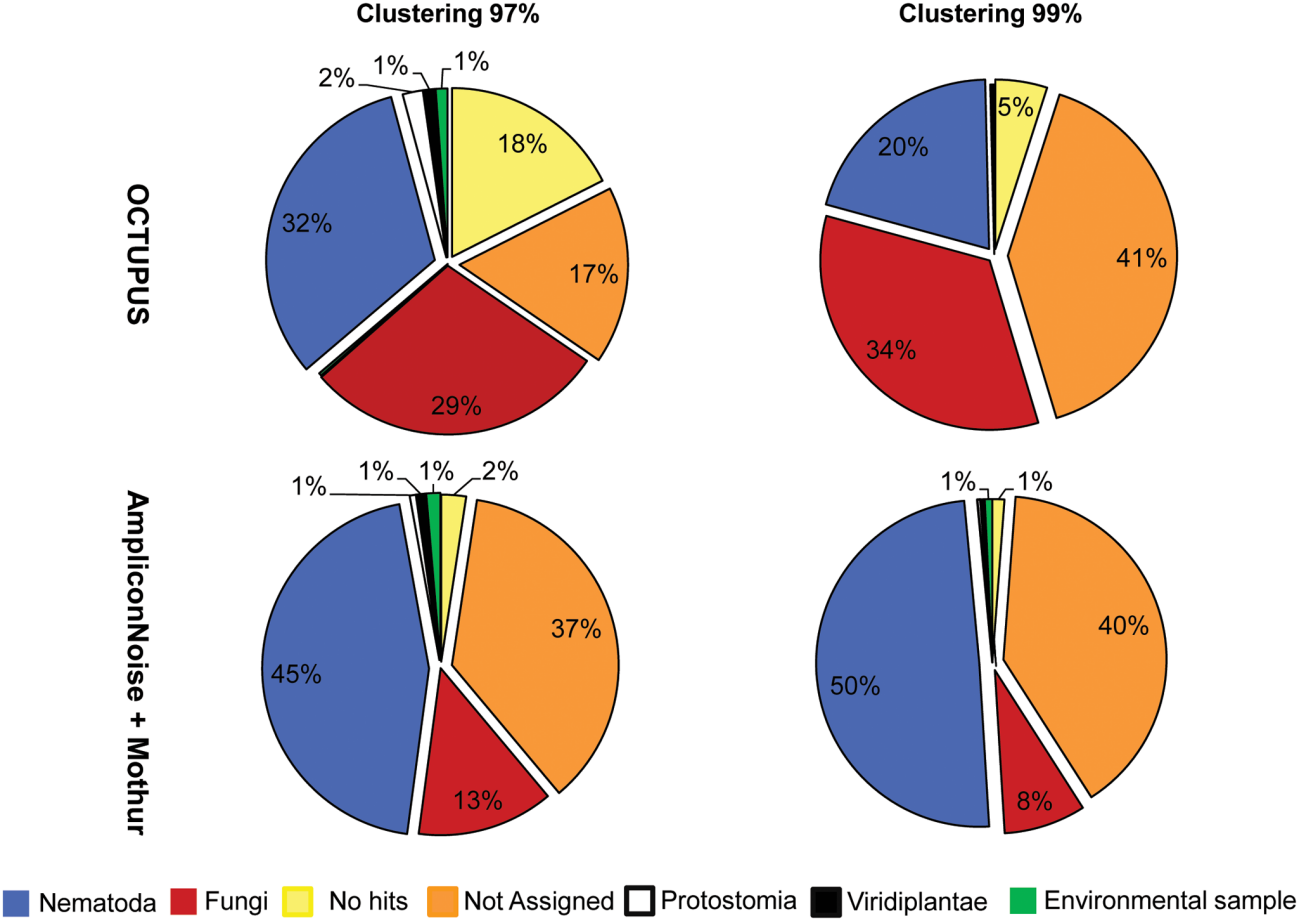
Eukaryotic taxa identified by metagenetic analysis of the nematode assemblage (10 individuals) collected in the NW Mediterranean Sea. Reported are the relative contribution of eukaryotic taxa obtained at 97% and 99% clustering thresholds using the OCTUPUS pipeline and AmpliconNoise plus Mothur programs. The contributions are calculated from non-chimeric Operational Clustered Taxonomic Units (OCTUs) representing forward sequencing reads from 18S rRNA gene. The contributions of the most important components (expressed as percentage) are reported.

Roche 454 sequencing generated a total of 56,851 reads (average length 355 bp) from the assemblage of 10 nematodes recovered from the NW Mediterranean Sea (Table 1). The number of OCTUs obtained after chimera removal and the number of chimeras are reported in Table 2. BLAST results against the Genbank database indicated the presence of OCTUs not only with hits for Nematoda, but also for Fungi, Plantae (i.e., Viridiplantae) and Protostomia (Fig 2). Especially with a clustering threshold of 97%, 115 OCTUs out of 359 (~ 32%) were assigned to the phylum Nematoda. The number of OCTUs assigned to nematodes decreased considerably when using the 99% cut-off (~ 20%) and the number of “not assigned” OCTUs (i.e., OCTUs with hits in the public database but without an assigned taxonomy; e.g., uncultured eukaryotes) increased (Fig 2; Table C in S1 File). Indeed, the contribution of “not assigned” OCTUs to the total OCTUs was 17% using 97% clustering threshold and 41% using 99%. A fraction of OCTUs (18% and ~5% for 97% and 99% clustering threshold, respectively) displayed no significant match (“no hit”, sequence identity <90%) to known ribosomal sequences (Fig 2; Table C in S1 File).

**Table 2:** Results of clustering analysis carried out with OCTUPUS and Mothur pipelines.

	% of clustering	OCTUs without chimeras	Chimeras
**OCTUPUS**			
10 nematodes (NW Mediterranean)	97%	359	325 (48%)
10 nematodes (NW Mediterranean)	99%	5356	1186 (18%)
100 nematodes (Central Mediterranean)	97%	1359	1151 (46%)
100 nematodes (Central Mediterranean)	99%	7128	2806 (28%)
**MOTHUR**			
10 nematodes (NW Mediterranean)	97%	666	345 (34%)
10 nematodes (NW Mediterranean)	99%	1346	345 (20%)
100 nematodes (Central Mediterranean)	97%	2096	1531 (42%)
100 nematodes (Central Mediterranean)	99%	3177	1553 (33%)

Clustering analyses have been carried out at 97% and 99% thresholds for nematode assemblages recovered from deep-sea sediments of the NW and Central Mediterranean Sea. In parenthesis the percentage of chimeras on the total OCTUs detected.

**Fig 3:**
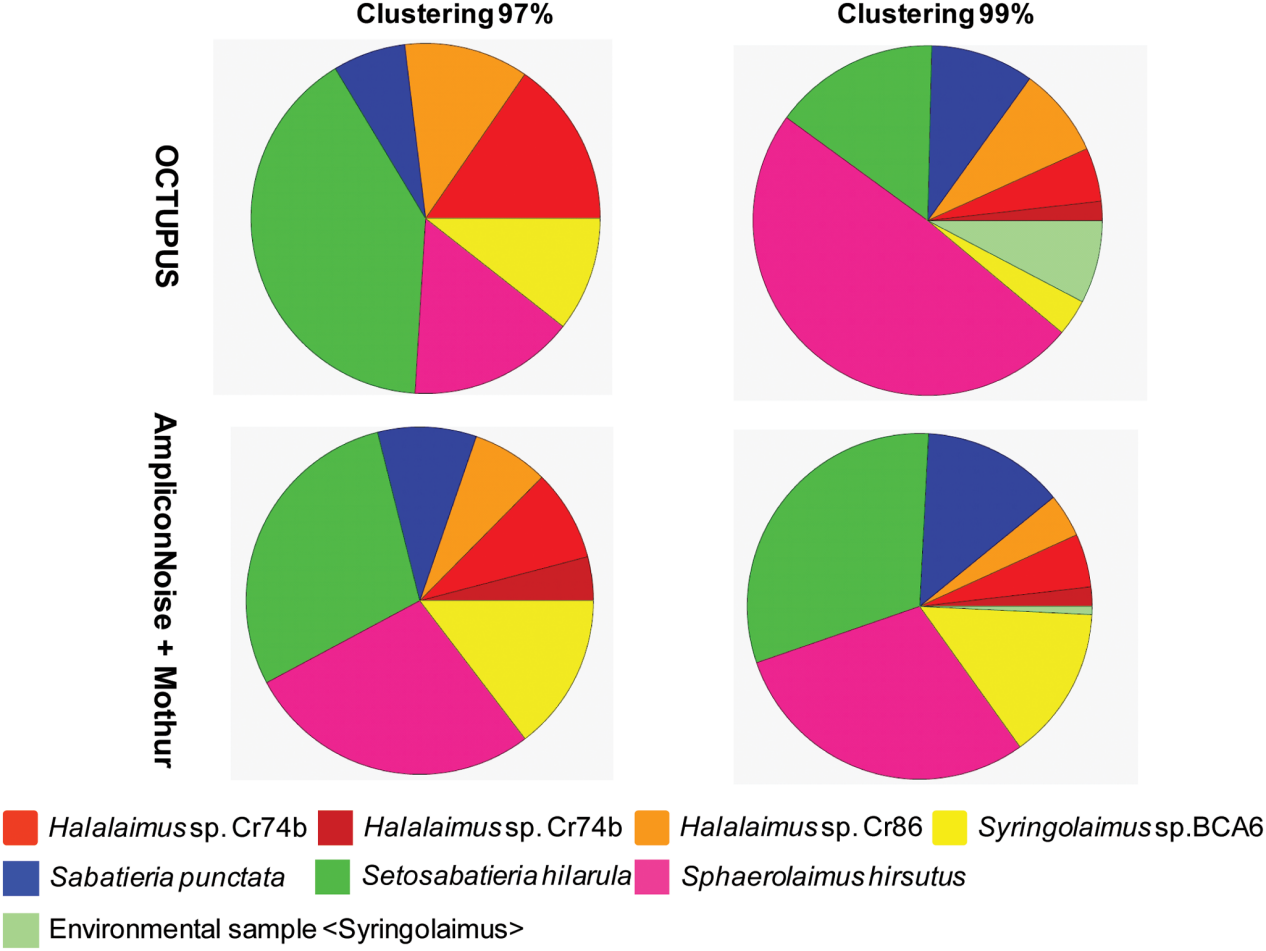
Relative proportion of OCTUs belonging to different nematode species within the nematode assemblage (10 individuals) collected in the NW Mediterranean Sea. Reported are the relative contribution of the different nematode genera obtained at 97% and 99% clustering thresholds using the OCTUPUS pipeline and AmpliconNoise plus Mothur programs. The contributions are calculated from non-chimeric OCTUs representing forward sequencing reads from 18S rRNA gene.

The analysis of OCTUs at the genus level (Fig 3) highlighted the presence of 5 out of 6 genera morphologically identified (the 18S rRNA of *Hopperia sp*. was lacking in the GenBank database) with a different contribution of OCTUs assigned to each genus depending on the clustering threshold used (Table D in S1 File). The relative contribution of OCTUs assigned to *Sabatieria* and *Sphaerolaimus* to the total OCTUs deviated significantly from the contribution of the individuals belonging to these two genera morphologically identified (Table 3). As far as the comparison at the species level is concerned (Fig 3), the molecular analysis allowed to identify two or three different species (for 97 and 99% clustering threshold, respectively) of *Halalaimus* (although not formally described), although only one morpho-species of *Halalaimus* was included in the nematode assemblage. On the contrary, by using the molecular approach only one species of *Sabatieria* out of four was identified. BLAST search analysis against the Genbank database of reads obtained using the Mothur pipeline indicated the presence of Fungi besides the Nematoda phylum, but the number of OCTUs assigned to this group was much lower (13 and 8% at 97% and 99% clustering threshold, respectively) with respect to the OCTUPUS results. Moreover, the taxonomic differences detected by OCTUPUS when using different clustering thresholds, were not so evident for reads analysed with Mothur (Fig 2). Considering a clustering threshold of 97%, 300 OCTUs (out of 666, 45%) were assigned to Nematoda, while at a 99% threshold 666 OCTUs (out of 1346, 50%) were identified as Nematoda. OCTUs labelled as “not assigned” represented also in this case a high proportion (37% and 40% at 97% and 99% clustering threshold), while “no hit” OCTUs decreased drastically (1–2%; Fig 2; Table C in S1 File). At the genus level OCTUs cleaned by using AmpliconNoise yielded a more faithful representation of the genera proportion derived from morphological identification. *Sabatieria* genus represented indeed 14% of the total nematode abundance, *Setosabatieria* sp. the 31%, *Sphaerolaimus* sp. the 29%, *Syringolaimus* sp. 15% and *Halalaimus* sp. 11%. These proportions were not significantly different from those obtained from morphological classification (χ^2^-test with Yates continuity correction, ns). However, the use of AmpliconNoise did not improve the identification at the species level, since only one species of *Sabatieria* out of the four morphologically identified were detected. Bioinformatic analyses performed using QIIME resulted in a lower number of genera (4 out of 6 genera morphologically identified) compared to that found using OCTUPUS and Mothur, at both 97% and 99% clustering thresholds.

**Table 3:** Comparison between molecular and morphological identification.

Genus	Molecular identification	Morphological identification	χ^2^-test 95% P-value
***Sabatieria***	10%	40%	**0.01**
***Setosabatieira***	15%	10%	0.994
***Sphaerolaimus***	48%	10%	**0.03**
***Syringolaimus***	11%	20%	0.682
***Halalaimus***	16%	10%	0.956
***Hopperia***	0%	10%	na

The relative contributions of OCTUs at 99% clustering threshold assigned to nematode genera by metabarcoding (using the OCTUPUS pipeline) and the relative contribution of individuals of the same genera identified by morphological analysis. The χ^2^-test has been performed with Yates continuity correction to evaluate the significance of the deviation between the two values. *Hopperia* sp. was not identified by sequencing. na = not applicable.

#### Analyses on deep-sea nematode assemblages from the Central Mediterranean Sea

**Fig 4:**
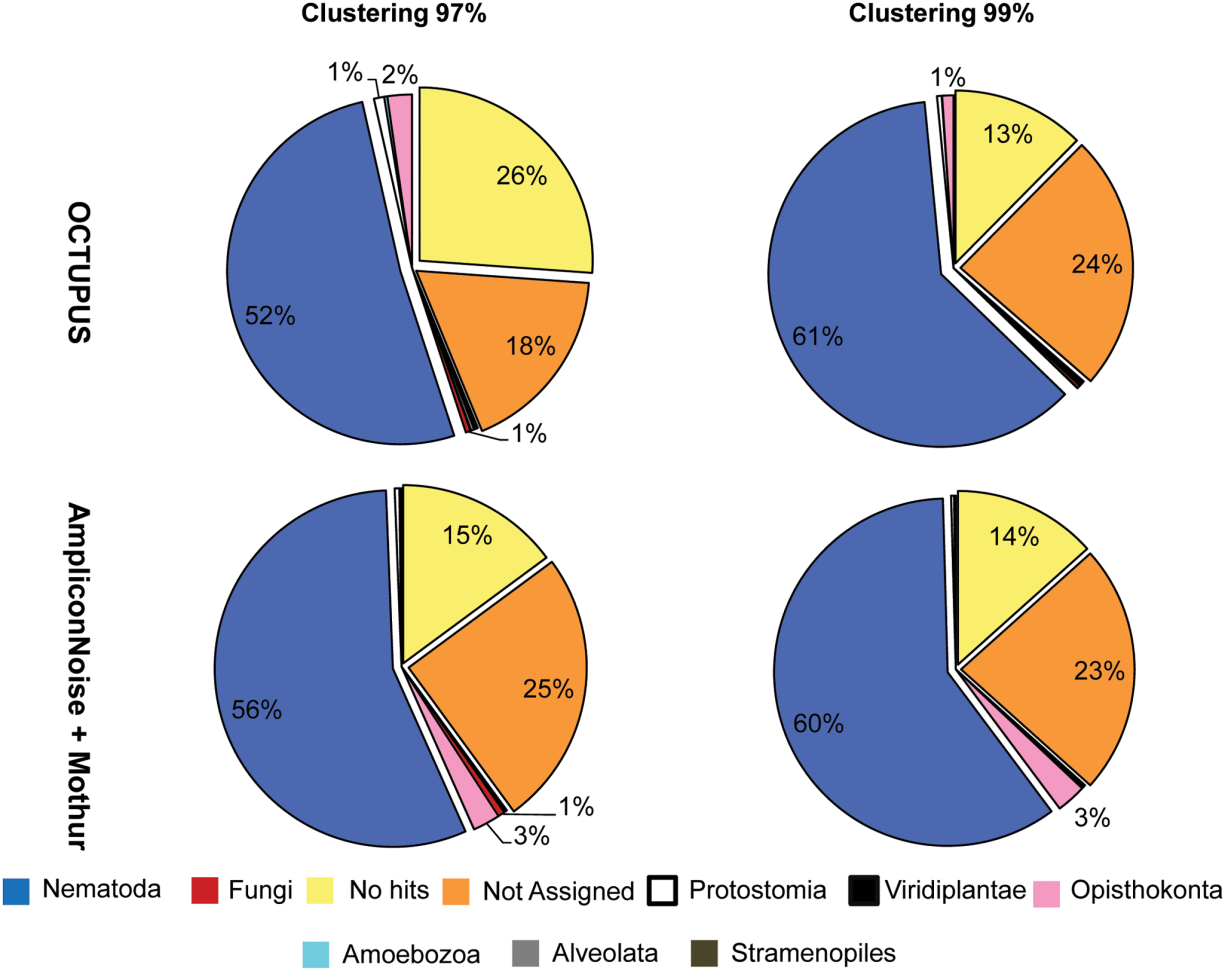
Eukaryotic taxa identified by metagenetic analysis of the nematode assemblage (100 individuals) collected in the Central Mediterranean Sea. Reported are the relative contribution of eukaryotic taxa obtained at 97% and 99% clustering thresholds using the OCTUPUS pipeline and AmpliconNoise plus Mothur programs. The contributions are calculated from non-chimeric Operational Clustered Taxonomic Units (OCTUs) representing forward sequencing reads from 18S rRNA gene. The contributions of the most important components (expressed as percentages) are reported.

Roche 454 sequencing analysis generated a total of 61,647 reads (average length 353bp) from the assemblage of 100 nematodes collected from the Central Mediterranean (Table 1). The number of OCTUs obtained by using OCTUPUS and Mothur pipelines after removing chimeras and the number of chimeras are reported in Table 2. By using OCTUPUS at 99% clustering threshold, 61% of OCTUs was assigned to Nematoda, whereas the number of OCTUs identified as both “not assigned” and “no hit” represented a relatively high percentage of the assemblage (24% and 13%, respectively; Fig 4; Table C in S1 File). At a 97% clustering threshold a higher percentage of “no hit” OCTUs was detected (26%; Fig 4), but the number of “not assigned” OCTUs was lower (18%; Fig 4; Table C in S1 File). Bioinformatic analysis of reads performed with the Mothur pipeline indicated that the percentage of organisms different from Nematoda (i.e., Fungi) was very low (ca. 1% at 97% and 99% clustering thresholds) whereas the percentages of “not assigned” and “no hit” OCTUs were 23–25% and 14–15%, respectively (Fig 4; Table C in S1 File). In general, the two different methods provided similar results in terms of assignment at higher taxonomy level (i.e. order).

**Fig 5:**
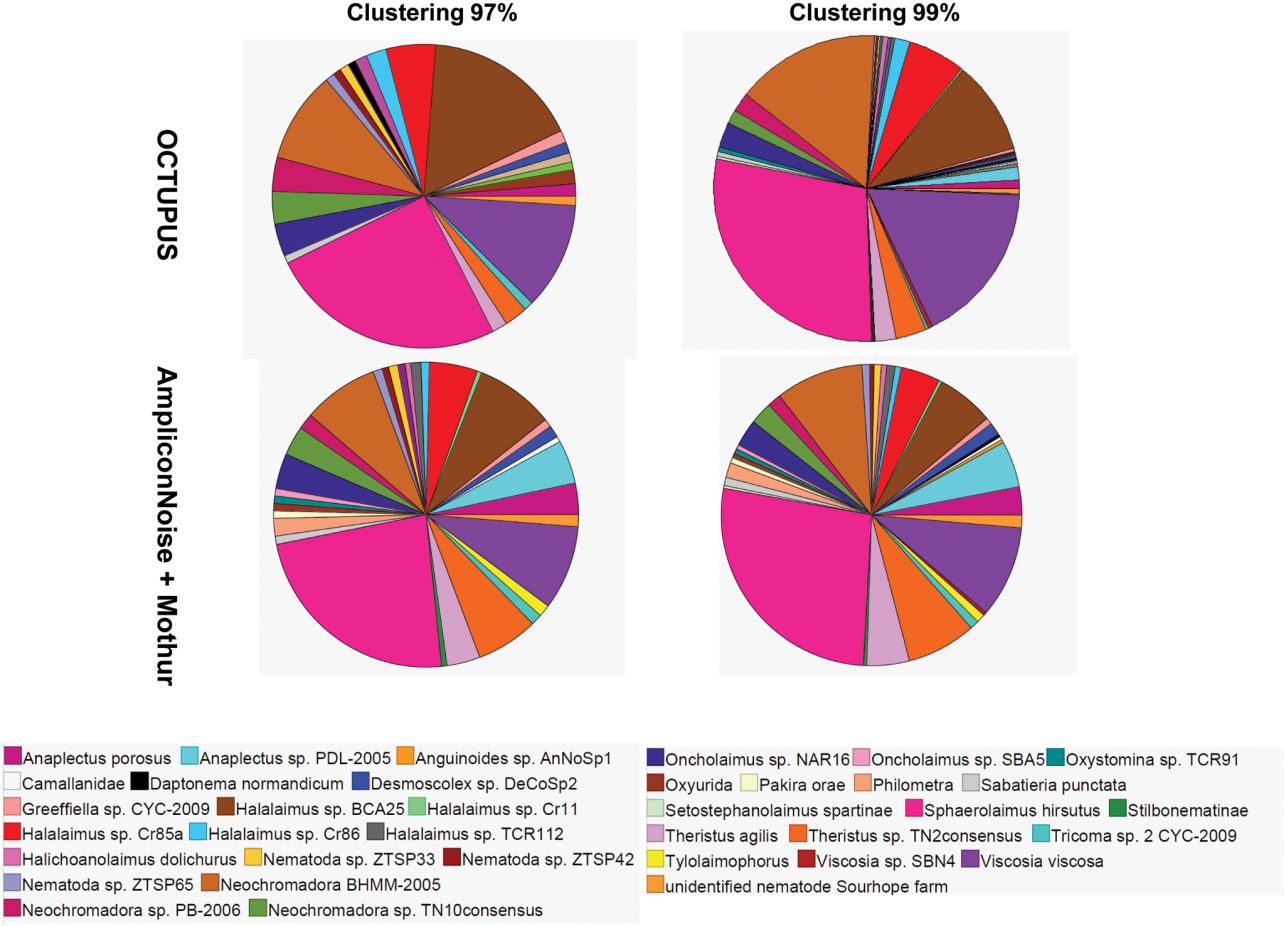
Relative proportion of OCTUs belonging to different nematode species within the nematode assemblage (100 individuals) collected in the Central Mediterranean Sea. Reported are the relative contribution of the different nematode species obtained at 97% and 99% clustering thresholds using the OCTUPUS pipeline and AmpliconNoise plus Mothur programs. The contributions are calculated from non-chimeric OCTUs representing forward sequencing reads from 18S rRNA gene.

Morphological analysis of nematodes collected in the Central Mediterranean allowed to identify 35 morpho-species (Table B in S1 File), whereas taxonomic identification of OCTUs performed with the MEGAN software allowed to retrieve 20 and 33 species using 97% and 99% clustering threshold, respectively by using OCTUPUS and 26 and 30 species using Mothur (Fig 5; Table D in S1 File). OCTUs obtained with OCTUPUS at 99% clustering threshold correctly detected 6 out of 6 nematode orders (with two further orders lacking using the morphological classification). OCTUs were classified in 19 families (Table D in S1 File) and 1 super-families, and 13 out of 17 families identified through the morphological analysis were properly assigned. Similarly the analysis at the 97% threshold allowed identifying the same orders detected with 99% threshold, but only 11 out of 17 families were assigned (Table D in S1 File). At 99% clustering threshold, 17 genera out of 27 were properly identified. The results obtained with Mothur provided comparable results: both at 97% and 99% clustering, 6 out 6 nematode orders were correctly identified (with three further orders lacking from morphological classification). OCTUs were classified in 12 and 14 families at 97% and 99% clustering threshold respectively (Table D in S1 File), and included 9 out of 17 families detected with the morphological analysis at both cut-offs. Using 99% clustering threshold, 14 genera out of 27 were correctly identified.

The bioinformatic analysis of reads performed using the QIIME pipeline produced a lower number of OCTUs (162 and 191 OCTUs at 97% and 99% cut-off, respectively) compared to that obtained using OCTUPUS and Mothur pipelines. Moreover, analyses using QIIME at 99% clustering threshold provided a lower number of genera (i.e., 16 genera) than using Mothur and OCTUPUS (i.e., 20 and 22 genera, respectively) by using the same taxonomic assignment method (e.g. GenBank BLAST match identity analysis and MEGAN [40]).

BLAST match analyses against the SILVA NR 119 database of OCTUs derived from the OCTUPUS pipeline provided a lower number of genera (i.e., 16 genera at 99% cut-off) compared to that found using the GenBank database (i.e., 22 genera at 99% cut-off; Table D in S1 File). BLAST match analyses against the SILVA NR 119 database of the OCTUs obtained through the Mothur pipeline detected the same number of genera as the GenBank database (i.e., 19 genera at 99% cut-off).

### Intra-genomic variability of 18S rRNA gene repeats of deep-sea nematodes

The total number of reads (average length 362 bp) obtained from Illumina MiSeq sequencing and the number of reads remained after trimming for each sequenced nematode morphospecies are reported in Table 1. The 97% and 99% clustering similarity thresholds allowed obtaining 15–102 and 18–108 OCTUs, respectively. The number of OCTUs assigned to the different morphospecies of deep-sea nematodes ranged from 1 to 5 at 97% and from 1 to 18 at 99% cut-off (Table 4).

**Table 4:** Results of intra-genomic variability analysis.

Morphospecies	OCTUs 97% clustering	OCTUs correctly assigned	OCTUs 99% clustering	OCTUs correctly assigned
***Halichoanolaimus sp1***	21	3	28	9
***Sabatieria sp1***	28	5	45	18
***Metachromadora sp1***	18	1	38	6
***Viscosia sp1***	15	1	18	1
***Thoracostoma sp*.**	102	3	108	5

Number of OCTUs at 97% and 99% clustering thresholds and number of OCTUs correctly assigned to each nematode morphospecies obtained using Illumina MiSeq.

## Discussion

### Methods for DNA extraction from marine nematodes suitable for molecular analysis

The recovery of DNA from meiofaunal organisms suitable for PCR amplification represents a crucial step for the assessment of their biodiversity based on molecular approaches. Previous molecular investigations carried out on marine nematodes, have used different DNA extraction procedures [17–20,23,24,45]. Some approaches are based on the use of an alkaline solution (NaOH) and freezing-thawing steps [21] and modifications [18]; others on the use of a lysis buffer containing proteinase K followed or not by DNA purification with commercial kits [10,17,20,46,47]. Further studies conducted on terrestrial nematodes have used commercial kits for the extraction and purification of DNA [26,27,30]. So far, the performance of these extraction procedures has never been compared, and this hampers the identification of the most suitable DNA extraction protocol for biodiversity analysis through molecular approaches. In this study different procedures were compared for their efficiency of DNA extraction from cultured and free-living nematodes collected from both coastal and deep-sea sediments. Our results indicate that the QIAGEN kit represents an efficient tool, among those tested, for DNA extraction from deep-sea nematodes. Moreover, 18S rDNA sequences, independently from the DNA extraction procedures used, were consistently obtained both in cultured and coastal nematodes. This was not the case for deep-sea nematodes, where only the QIAGEN and MoBio kits provided always 18S rDNA amplicons which can be sequenced by using both Sanger and HTS. This could be explained considering that PCR amplification efficiency relies from one side on the amount of DNA template and from the other from its purity (i.e. lack of inhibitors for polymerase reactions). Therefore, these results indicate that the use of these commercial kits allow us to recover an amount of DNA from marine nematodes free of potential inhibitors, suitable for amplification and as such they should be preferred for the molecular analysis of biodiversity of deep-sea nematodes.

### Identification of deep-sea nematodes based on metagenetics

In the present study we found that different cut-offs for OCTUs generation (i.e. a different level of similarity for the identification of OCTUs) using the OCTUPUS pipeline provided different estimates of taxon richness. This is not surprising since the intra-specific variation can be detected using more stringent thresholds (99%), whereas genera and order can be grouped using more conservative thresholds (97%) [48]. Such differences in taxon richness between the two clustering thresholds were not so evident using Mothur program. These findings suggest that the use of 99% cut-off provides a better resolution in assigning sequences to the species level [26] for deep-sea nematodes.

Our results pointed out also that the number of OCTUs for each genus did not reflect the number of individuals belonging to the same genus morphologically identified in the sample. In particular, for the assemblage of 10 nematodes, by using the OCTUPUS pipeline, two out of five genera (*Sabatieria* and *Sphaerolaimus*) showed a significant deviation from the expected quantitative representation. Different reasons related to PCR amplification can be invoked to explain such differences, including: i) differences in the amplification efficiency of the different genera, which can lead to a different proportion of OCTUs with respect to the number of individuals per genus; ii) occasional failure of nematode templates due to the use of primers that are highly conserved in eukaryotes and iii) loss of some amplicons during the emulsion PCR step [23]. At the same time, it should be taken into account that the four specimens belonging to the *Sabatieria* genus and identified as morphologically different could be only phenotypically diverse, but genetically identical. Despite this, by using AmpliconNoise and Mothur none of these proportions significantly deviated from the expected values suggesting that the cleaning of sequences before the analysis can reduce the gap between proportions obtained from morphological and molecular analysis. Moreover, we found that using 99% clustering threshold a high number of OCTUs were attributed to *Sphaerolaimus* and *Setosabatieria* despite they were represented by only one individual in the nematode assemblage. These findings suggest either a potential incorrect assignment of sequences belonging to other genera (i.e. *Hopperia sp*.), the presence of intra-individual variation among rRNA gene copies, or errors occurring during amplification and/or sequencing steps.

The taxonomic identification of the OCTUs proceeds through a similarity analysis against public databases, so that the output depends on nematode sequences deposited therein. Indeed, our results show that the number of genera obtained from Roche 454 sequencing analysis of DNA recovered from the nematode assemblage of the NW Mediterranean Sea was lower than that morphologically identified (as 18S rDNA sequences of the genus *Hopperia* are lacking in the public databases). Moreover, a high proportion of OCTUs (41% and 40% at 99% clustering threshold for OCTUPUS and Mothur analysis, respectively) displayed a significant match with unclassified ribosomal sequences (“Not assigned”). Roche 454 sequencing analysis carried out on the nematode assemblage of 100 individuals, provides further evidence of the limits of similarity search tools against databases, which, at present, contain a very low number of marine nematode sequences classified with no ambiguities (i.e., assigned to a genus or species instead of to a general “unknown nematode”). At the same time, the lack of taxonomic resolution at the species level of most marine nematodes (i.e. morphologically classified at the genus level followed by generic labels for the discrimination at species level, i.e. sp.1, sp.2 …) represents an additional problem for the correct assignment of nematode sequences. There is thus a strong need to improve the information contained in public databases linking DNA sequences to formally described species.

### Influence of polymorphisms on nematode biodiversity estimates

In the present study the total number of OCTUs assigned to nematodes was considerably higher than the actual number of individuals. This discrepancy could be due to the variability of 18S rRNA gene within nematode species and/or amplification and sequencing errors. Indeed, we found a variable number of OCTUs for the five morphospecies of deep-sea nematodes analysed (up to 5 and 18 using 97 and 99% cut-offs, respectively). Similarly, previous studies reported that the number of OCTUs matching each nematode species extracted from soils varies from 2 to 95 (using 99% cut-off) [31]. Our results suggest that nematodes inhabiting benthic deep-sea ecosystems can be characterised by the presence of polymorphisms among rRNA gene copies within their genomes, as also reported from whole genome shotgun library analyses carried out on different terrestrial nematode species [32]. Further studies are needed to elucidate the extent of the polymorphism of 18S rRNA gene in different marine nematode species and among different individuals of the same species. However, polymorphisms do not allow us to completely explain the OCTUs number we found using Roche 454 sequencing analysis, which could be further enhanced by PCR and sequencing errors. Indeed, it is known that Roche 454 is more prone to errors than Illumina MiSeq (1% *vs*. 0.1% per base within single reads; [37]). The advancement of HTS platforms along with the possibility to avoid the amplification step will allow us to reduce the errors and thus to provide more confident estimates of the actual OCTU number for each species.

## Conclusions

There is increasing evidence that deep-sea ecosystems are highly vulnerable to biodiversity losses, which can have major adverse consequences for key ecological processes (i.e., nutrient regeneration, biomass production) [2,4]. Since deep-sea ecosystems are already threatened by human impact through trawling, dumping, oil, gas and mineral extraction, and other pollution sources [49], there is an urgent need to develop suitable tools to be used for the quantification of the actual biodiversity in the deep sea. Our results indicate that the use of metabarcoding allows the analysis of the diversity of complex communities in a rapid and cost-effective way, thus increasing enormously our ability to investigate deep-sea nematode biodiversity. However, the identification of nematodes at genus and species level is still problematic, due to the limited number of sequences deposited in public databases. Thus we still need to couple morphological identification and the sequencing of the 18S rRNA gene (typically using Sanger sequencing) to implement the available databases. Additional efforts are also needed to understand the actual variability of the 18S rRNA gene repeats among marine nematodes and to identify alternative single copy markers (nuclear or mitochondrial) able to provide quantitative estimates of the relative contribution of each species to the whole assemblage. These aspects should be carefully taken into account for using metabarcoding in quantitative ecological research and monitoring programmes of marine biodiversity.

## Supporting Information

S1 File Details on PCR analysis **(Appendix)**. List of morpho-species belonging to different genera of nematodes (10 individuals) collected in deep-sea sediments of the NW Mediterranean Sea utilised for metagenetic analysis **(Table A)**. List of morpho-species belonging to different genera of nematodes (100 individuals) collected in deep-sea sediments of the Central Mediterranean Sea utilised for metagenetic analysis **(Table B)**. Number of OCTUs classified as “Not Assigned” and “No hit” after BLAST Search using OCTUPUS and Mothur pipelines at 97% and 99% clustering thresholds **(Table C)**. Comparison between the total number of families, genera and species identified by using the morphological criteria and the metagenetic approach within the nematode assemblages recovered from the NW and Central Mediterranean Sea **(Table D)**. Gel electrophoresis of PCR products using the primer pairs Nem_18S_F and Nem_18S_R from DNA of nematodes collected from benthic shallow-water systems **(Figure A)**. Gel electrophoresis of PCR products using the primer pairs Nem_18S_F and Nem_18S_R from DNA of deep-sea nematodes (*Sphaerolaimus uncinatus*) extracted with NaOH procedure and the QIAGEN kit **(Figure B)**. Gel electrophoresis of PCR products using the primer pairs SSUF04 and SSUR22 from DNA of nematodes collected from deep-sea sediments **(Figure C)**.
(DOC)
