# Supplementary material for: Unveiling the Biodiversity of Deep-Sea Nematodes through Metabarcoding: Are We Ready to Bypass the Classical Taxonomy?
Source: PLoS One. 2015 Dec 23;10(12):e0144928. doi: 10.1371/journal.pone.0144928 (PMC4699195; doi:10.1371/journal.pone.0144928)
Supplement: S1 File — Details on PCR analysis (Appendix). List of morpho-species belonging to different genera of nematodes (10 individuals) collected in deep-sea sediments of the NW Mediterranean Sea utilised for metagenetic analysis (Table A). List of morpho-species belonging to different genera of nematodes (100 individuals) collected in deep-sea sediments of the Central Mediterranean Sea utilised for metagenetic analysis (Table B). Number of OCTUs classified as “Not Assigned” and “No hit” after BLAST Search using OCTUPUS and Mothur pipelines at 97% and 99% clustering thresholds (Table C). Comparison between the total number of families, genera and species identified by using the morphological criteria and the metagenetic approach within the nematode assemblages recovered from the NW and Central Mediterranean Sea (Table D). Gel electrophoresis of PCR products using the primer pairs Nem_18S_F and Nem_18S_R from DNA of nematodes collected from benthic shallow-water systems (Figure A). Gel electrophoresis of PCR products using the primer pairs Nem_18S_F and Nem_18S_R from DNA of deep-sea nematodes (Sphaerolaimus uncinatus) extracted with NaOH procedure and the QIAGEN kit (Figure B). Gel electrophoresis of PCR products using the primer pairs SSUF04 and SSUR22 from DNA of nematodes collected from deep-sea sediments (Figure C). (DOC) [file pone.0144928.s001.doc]

Supporting Information

**Unveiling the biodiversity of deep-sea nematodes through metabarcoding: are we ready to bypass the classical taxonomy?**

Antonio Dell’Anno, Laura Carugati, Cinzia Corinaldesi, Giulia Riccioni, Roberto Danovaro

**Appendix**

PCR reactions using the primer pairs Nem_18S_F and Nem_18S_R were carried out in a total volume of 50 µL containing 50-100 pg DNA as template, 0.4 µM of each primer, 0.05 Units µL^-1^ MyTAQ™ HS DNA Polymerase (Bioline), 1× MyTAQ™ HS reaction buffer (including 3 mM MgCl_2_), 1 mM dNTPs and 0.5mg ml^-1^ BSA (Applied Biosystems). The adopted thermal cycling conditions were: an initial denaturation at 95°C for 2 min, 35 cycles of amplification (30 s at 94°C, 1,30 min at 54°C, 3 min at 72°C) followed by a final extension at 72°C for 10 min. Negative controls were included for each amplification reaction. All PCR reactions were done in a Veriti thermal cycler (Applied Biosystems) and PCR products of the expected length size (i.e. 900 bp) were checked using 1% agarose gel electrophoresis after staining with GelRed (BIOTIUM). PCR products once purified using ExoSAP-IT (Affimetrix) were also sequenced by Sanger chain-termination sequencing. PCR reactions using the primer pairs SSUF04 and SSUR22 were carried out in a total volume of 50 µL containing 50-100 pg DNA as template, 0.4 µM of each primer, 0.04 Units µL^-1^ PrimeSTAR GXL DNA polymerase (Takara), 1× PrimeSTAR reaction buffer (including 3 mM MgCl_2_) and 0.8 mM dNTPs. The adopted thermal cycling conditions were similar to those previously published [1], with slight modifications. After an initial denaturation at 94°C for 10 min, 35 cycles of amplification (1 min. at 94°C, 1,30 min at 57°C, 40 sec at 72°C) followed by a final extension at 72°C for 10 min have been used. Negative controls were included for each amplification reaction. PCR products were checked using 1% agarose gel electrophoresis after staining with GelRed (BIOTIUM).

**Table A.** List of morpho-species belonging to different genera of nematodes (10 individuals) collected in deep-sea sediments of the NW Mediterranean Sea utilised for metagenetic analysis. ID= identification number

| **ID** | **Morpho-species** | **ID** | **Morpho-species** |
| --- | --- | --- | --- |
| **1** | *Sabatieria sp. 1* | **6** | *Syringolaimus sp. 2* |
| **2** | *Sabatieria sp. 2* | **7** | *Halalaimus sp. 1* |
| **3** | *Sabatieria sp. 3* | **8** | *Sphaerolaimus sp. 1* |
| **4** | *Sabatieria sp. 4* | **9** | *Setosabatieria sp. 1* |
| **5** | *Syringolaimus sp. 1* | **10** | *Hopperia sp. 1* |

**Table B** List of morpho-species belonging to different genera of nematodes (100 individuals) collected in deep-sea sediments of the Central Mediterranean Sea utilised for metagenetic analysis. ID= identification number

| **ID** | **Morpho-species** | **ID** | **Morpho-species** | **ID** | **Morpho-species** | **ID** | **Morpho-species** |
| --- | --- | --- | --- | --- | --- | --- | --- |
| **1** | *Halalaimus sp. 1* | **26** | *Desmodora sp. 5* | **51** | *Desmoscolex sp. 4* | **76** | *Sphaerolaimus sp. 1* |
| **2** | *Desmoscolex sp. 1* | **27** | *Richtersia sp. 5* | **52** | *Desmoscolex sp. 2* | **77** | *Desmoscolex sp. 4* |
| **3** | *Halalaimus sp. 9* | **28** | *Pselionema sp. 1* | **53** | *Sabatieria sp. 1* | **78** | *Halichoanolaimus*  *sp. 4* |
| **4** | *Halalaimus sp. 9* | **29** | *Viscosia sp. 1* | **54** | *Sphaerolaimus sp. 1* | **79** | *Parasphaerolaimus sp. 1* |
| **5** | *Halalaimus sp. 9* | **30** | *Viscosia sp. 1* | **55** | *Leptolaimus sp. 5* | **80** | *Desmodora sp. 5* |
| **6** | *Sphaerolaimus sp. 1* | **31** | *Sphaerolaimus sp. 1* | **56** | *Monhystrella* | **81** | *Halalaimus sp. 9* |
| **7** | *Halichoanolaimus sp. 1* | **32** | *Cyatholaimus sp. 1* | **57** | *Theristus sp. 1* | **82** | *Tricoma sp. 1* |
| **8** | *Tricoma sp. 1* | **33** | *Tricoma sp. 5* | **58** | *Desmoscolex sp. 4* | **83** | *Halalaimus sp. 1* |
| **9** | *Parasphaerolaimus sp. 1* | **34** | *Tricoma sp. 1* | **59** | *Syringolaimus filicaudatus* | **84** | *Halalaimus sp. 9* |
| **10** | *Sabatieria sp. 1* | **35** | *Desmodora sp. 1* | **60** | *Neochromadora*  *sp. 1* | **85** | *Richtersia sp. 2* |
| **11** | *Procamacolaimus sp. 1* | **36** | *Anaplectus* | **61** | *Halalaimus sp. 1* | **86** | *Sphaerolaimus sp. 1* |
| **12** | *Procamacolaimus sp. 1* | **37** | *Bathyeurystomina sp. 1* | **62** | *Daptonema sp. 1* | **87** | *Tricoma sp. 1* |
| **13** | *Molgolaimus sp. 1* | **38** | *Sphaerolaimus sp. 1* | **63** | *Tricoma sp. 1* | **88** | *Daptonema sp. 1* |
| **14** | *Molgolaimus sp. 1* | **39** | *Oncholaimus* | **64** | *Pselionema sp. 3* | **89** | *Desmoscolex sp. 4* |
| **15** | *Procamacolaimus sp. 1* | **40** | *Pselionema sp. 3* | **65** | *Tricoma sp. 1* | **90** | *Richtersia sp. 2* |
| **16** | *Sabatieria sp. 1* | **41** | *Desmoscolex sp. 1* | **66** | *Tricoma sp. 1* | **91** | *Daptonema sp. 1* |
| **17** | *Desmodora sp. 1* | **42** | *Greeffiella* | **67** | *Spilophorella sp. 1* | **92** | *Desmoscolex sp. 4* |
| **18** | *Desmoscolex sp. 2* | **43** | *Theristus sp. 1* | **68** | *Desmoscolex sp. 4* | **93** | *Richtersia sp. 2* |
| **19** | *Sphaerolaimus sp. 1* | **44** | *Sphaerolaimus sp. 1* | **69** | *Parasphaerolaimus sp. 1* | **94** | *Spilophorella sp. 1* |
| **20** | *Tricoma sp. 1* | **45** | *Tricoma sp. 1* | **70** | *Desmoscolex sp. 4* | **95** | *Spilophorella sp. 1* |
| **21** | *Parasphaerolaimus sp. 1* | **46** | *Spilophorella sp. 1* | **71** | *Tricoma sp. 1* | **96** | *Richtersia sp. 2* |
| **22** | *Sphaerolaimus sp. 1* | **47** | *Oxystomina sp. 1* | **72** | *Sabatieria sp. 1* | **97** | *Richtersia sp. 2* |
| **23** | *Microlaimus sp. 3* | **48** | *Richtersia sp. 5* | **73** | *Spilophorella sp. 1* | **98** | *Tricoma sp. 1* |
| **24** | *Richtersia sp. 5* | **49** | *Desmodora sp. 1* | **74** | *Halichoanolaimus sp. 4* | **99** | *Desmoscolex sp. 4* |
| **25** | *Microlaimus sp. 3* | **50** | *Halalaimus sp. 9* | **75** | *Spilophorella sp. 1* | **100** | *Richtersia sp. 2* |

**Table C.** Number of OCTUs classified as “Not Assigned” and “No hit” after BLAST Search using OCTUPUS and Mothur pipelines at 97% and 99% clustering thresholds. In parenthesis the percentage on the total number of OCTUs.

|  | **“Not Assigned” OCTUs** | **“No hit” OCTUs** |
| --- | --- | --- |
| **OCTUPUS** |  |  |
| 10 nematodes - 97% clustering | 61 (17%) | 63 (18%) |
| 10 nematodes - 99% clustering | 2164 (41%) | 265 (5%) |
| 100 nematodes - 97% clustering | 240 (18%) | 355 (26%) |
| 100 nematodes - 99% clustering | 1709 (24%) | 885 (13%) |
| **MOTHUR** |  |  |
| 10 nematodes - 97% clustering | 243 (37%) | 16 (2%) |
| 10 nematodes - 99% clustering | 535 (40%) | 16 (1%) |
| 100 nematodes - 97% clustering | 525 (25%) | 312 (15%) |
| 100 nematodes - 99% clustering | 739 (23%) | 425 (14%) |

**Table D. Comparison between the total number of families, genera and species identified by using the morphological criteria and the metagenetic approach within the nematode assemblages recovered from the NW and Central Mediterranean Sea. The results of the three different bioinformatic pipelines and two different cut-off values are reported.**

|  |  |  |  | **10 deep-sea nematodes from NW Mediterranean Sea** | **100 deep-sea nematodes from Central Mediterranean Sea** |
| --- | --- | --- | --- | --- | --- |
| **Morphological identification** |  |  | Families | 4 | 17 |
|  |  |  | Genera | 6 | 27 |
|  |  |  | Species | 10 | 35 |
|  | Pipeline | Cut-off  (%) |  |  |  |
| **Molecular identification** | OCTUPUS | 97 | Families | 4 | 11 |
|  |  | 99 |  | 4 | 19 |
|  |  | 97 | Genera | 5 | 11 |
|  |  | 99 |  | 5 | 22 |
|  |  | 97 | Species | 6 | 20 |
|  |  | 99 |  | 8 | 33 |
|  | Mothur | 97 | Families | 4 | 12 |
|  |  | 99 |  | 4 | 14 |
|  |  | 97 | Genera | 5 | 17 |
|  |  | 99 |  | 5 | 20 |
|  |  | 97 | Species | 7 | 26 |
|  |  | 99 |  | 8 | 30 |
|  | QIIME | 97 | Families | 4 | 12 |
|  |  | 99 |  | 4 | 12 |
|  |  | 97 | Genera | 4 | 16 |
|  |  | 99 |  | 4 | 16 |
|  |  | 97 | Species | 5 | 22 |
|  |  | 99 |  | 6 | 22 |

**Figure A.** Gel electrophoresis of PCR products using the primer pairs Nem_18S_F and Nem_18S_R from DNA of nematodes collected from benthic shallow-water systems. Lane M: molecular marker (BIOLINE, 2.5 Kbp); lanes 1-2: 18S rRNA amplicons obtained from DNA of nematodes extracted with the QIAGEN kit; lanes 3-4: 18S rRNA amplicons obtained from DNA of nematodes extracted with NaOH procedure; lane 5: negative control.

**Figure B**. Gel electrophoresis of PCR products using the primer pairs Nem_18S_F and Nem_18S_R from DNA of deep-sea nematodes (*Sphaerolaimus uncinatus*) extracted with NaOH procedure (1) and QIAGEN kit (2). (1) Lane M: molecular marker (BIOLINE, 2.5 Kbp); lanes 1-10: 18S rRNA amplicons of *Sphaerolaimus uncinatus*, lane 11: positive control (obtained from PCR analysis of DNA from shallow water nematodes), lane 12: negative control. (2) Lane M: molecular marker (BIOLINE, 2.5 Kbp), lanes 1-7: 18S rRNA amplicons of *Sphaerolaimus uncinatus*, lane 8: positive control (obtained from PCR analysis of DNA from shallow water nematodes), Lane 9: negative control.

**Figure C**. Gel electrophoresis of PCR products using the primer pairs SSUF04 and SSUR22 from DNA of nematodes collected from deep-sea sediments. Lane M: molecular marker (BIOLINE, 2.5 Kbp); lanes 1-2: 18S rRNA amplicons obtained from DNA of nematodes extracted with the QIAGEN kit; lanes 3-5: 18S rRNA amplicons obtained from DNA of nematodes extracted with MoBio kit; lane 6: positive control (obtained from PCR analysis of DNA from shallow water nematodes); lane 7: negative control.

**References**

1. Fonseca VG, Carvalho GR, Sung W, Johnson HF, Power DM, Neill SP, et al. (2010) Second-generation environmental sequencing unmasks marine metazoan biodiversity. Nat Commun 1**:** 98.
